# Bushenkangshuai Tablet Reduces Atherosclerotic Lesion by Improving Blood Lipids Metabolism and Inhibiting Inflammatory Response via TLR4 and NF-*κ*B Signaling Pathway

**DOI:** 10.1155/2018/1758383

**Published:** 2018-01-24

**Authors:** Shu-chao Pang, Li Peng, Jun-ping Zhang, Yuan-yuan Wang, Hui-yun Jia, Li-yuan Bi, Mei-ling Chen

**Affiliations:** ^1^First Teaching Hospital of Tianjin University of Traditional Chinese Medicine, Tianjin 300193, China; ^2^Tianjin University of Traditional Chinese Medicine, Tianjin 300193, China; ^3^Medical College, Xiamen University, Xiamen 361102, China

## Abstract

Bushenkangshuai tablet (BSKS) is a Chinese herbal compound which has been used for the treatment of cardiovascular and cerebrovascular diseases in China for decades. This study intends to explore the molecular mechanism of BSKS against atherosclerosis in ApoE^−/−^ mice. ApoE^−/−^ mice were fed with western-type diet for 6 weeks and then were given BSKS for 6 weeks. The results showed that BSKS attenuated the size of the atherosclerotic lesion, reduced visceral adipose content, and decreased blood lipids. We also found that BSKS promoted the expression of adiponectin and its receptors, inhibited the expression of Toll-like receptor 4 and nuclear factor-kappa B, decreased the levels of interleukin-1 beta, monocyte chemotactic protein-1, and vascular cell adhesion molecule-1, and increased the levels of interleukin-10 and adiponectin. Our data provided evidence that BSKS exerted an antiatherosclerotic effect by lowering blood lipids and inhibiting inflammatory response via TLR4 and NF-*κ*B signaling pathway.

## 1. Introduction

Atherosclerosis (AS) is the main pathological foundation of cardiovascular and cerebrovascular diseases and is considered as an inflammatory disease [1]. It is well known that obesity contributes to the increased risk of cardiovascular and cerebrovascular diseases [2]. The visceral adipose tissue secretes several adipocytokines, such as adiponectin, leptin, and tumor necrosis factor-*α* (TNF-*α*), which has been proved to reduce or increase atherosclerosis [3, 4]. Increasing evidence shows that adiponectin exerts an important antiatherogenic effect by reducing the levels of adhesion molecules, preventing TNF-*α*-induced activation of nuclear factor-kappa B (NF-*κ*B), inhibiting the proliferation and migration of smooth muscle cells, and decreasing foam cell formation and platelet aggregation [5–7]. What is more, a study has confirmed that adiponectin inhibits Toll-like receptor- (TLR-) mediated NF-*κ*B signaling in mouse macrophages [8]. These effects of adiponectin not only come from systemic secretion, but also might be due to a local secretory pathway, the perivascular adipose tissue [9].

Bushenkangshuai tablet (BSKS) is a Chinese herbal compound which has been used to treat cardiovascular diseases in clinics [10, 11]. BSKS is composed of* Salvia miltiorrhiza *Bunge (the root of red-rooted* Salvia*, Danshen),* Reynoutria multiflora *(Thunb.) Moldenke (*Polygonum multiflorum*, Heshouwu),* Poria cocos *(Schw.) Wolf. (*Poria cocos*, Fuling),* Sargassum pallidum *(Turn.) C. Ag. (seaweed, Haizao),* Ecklonia kurome *Okam. (Laminaria, Kunbu),* Taxillus sutchuenensis *(Lecomte) Danser (parasitic* Loranthus*, Sangjisheng),* Eucommia ulmoides *Oliv. (the bark of* Eucommia*, Duzhong),* Cuscuta chinensis *Lam. (the seed of Chinese dodder, Tusizi), Testudinis Carapax et Plastrum (tortoise shell, Guijia),* Acorus calamus *var.* angustatus *Besser (*Acorus gramineus* Soland., Shichangpu),* Prunella vulgaris *L. (self-heal, Xiakucao),* Ligusticum striatum *DC. (*Ligusticum wallichii*, Chuanxiong),* Citrus aurantium *L. (dried tangerine or orange peel, Chenpi),* Cinnamomum cassia *(L.) J. Presl (cinnamon, Rougui), and* Codonopsis pilosula *(Franch.) Nannf. (*Codonopsis pilosula*, Dangshen). Studies have shown that* Salvia miltiorrhiza *Bunge is reported to accelerate macrophage cholesterol efflux by targeting peroxisome proliferator-activated receptor gamma and liver X receptor alpha [12, 13].* Reynoutria multiflora *(Thunb.) Moldenke and* Poria cocos *(Schw.) Wolf. have been suggested to have an antihyperlipidemic effect in diet-induced hyperlipidemia [14, 15].* Sargassum pallidum *(Turn.) C. Ag. is reported to decrease monocyte adhesion to endothelial cells by inhibiting TNF-*α*-induced NF-*κ*B signaling [16].* Taxillus sutchuenensis *(Lecomte) Danser decreases NO production in lipopolysaccharides-induced RAW 264.7 cells [17].* Eucommia ulmoides *Oliv. and* Citrus aurantium *L. have an anti-inflammatory activity [18, 19], and* Cuscuta chinensis *Lam. and* Prunella vulgaris *L. have antioxidant effects [20, 21]. Our previous study has verified that BSKS could reduce experimental atherosclerosis in rabbits by inhibiting the NF-*κ*B signaling pathway and inflammatory factors [22]. In this study, we intend to further investigate the molecular mechanism of BSKS against atherosclerosis in ApoE^−/−^ mice.

## 2. Materials and Methods

### 2.1. Animals

Eight-week-old male C57BL/6J mice and ApoE^−/−^ mice with a C57BL/6J background weighing 20–22 g were obtained from Beijing Huafukang Bioscience Co., Ltd. (Beijing, China) (certificate number SCXK (Jing) 2014-0004) [23]. Mice were housed in groups (4/cage) at 21–24°C and 41–62% relative humidity under a 12 h light-dark cycle and fed with standard diet and water freely. The animal protocols of this study were performed according to the National Institutes of Health's* Guide for the Care and Use of Laboratory Animals* [24] and were approved by the Animal Ethics Committee of Tianjin University of Traditional Chinese Medicine (Tianjin, China).

### 2.2. Drugs

Bushenkangshuai tablet (cat. number TJZB-Z2008110052, specification: 0.5 g/tablet) was provided by the Pharmacy Department of the First Teaching Hospital of Tianjin University of Traditional Chinese Medicine (Tianjin, China), which was produced based on the guidelines of Good Manufacturing Practice and Good Laboratory Practice. Its quality control conformed to the medical institutions' standards of Tianjin Food and Drug Administration. BSKS was composed of 15 components in total:* Salvia miltiorrhiza *Bunge,* Reynoutria multiflora *(Thunb.) Moldenke,* Poria cocos *(Schw.) Wolf.,* Sargassum pallidum *(Turn.) C. Ag.,* Ecklonia kurome *Okam.,* Taxillus sutchuenensis *(Lecomte) Danser,* Eucommia ulmoides *Oliv.,* Cuscuta chinensis *Lam., Testudinis Carapax et Plastrum,* Acorus calamus *var.* angustatus *Besser,* Prunella vulgaris *L.,* Ligusticum striatum *DC.,* Citrus aurantium *L.,* Cinnamomum cassia *(L.) J. Presl, and* Codonopsis pilosula *(Franch.) Nannf. The proportion of each component in BSKS was equal. Atorvastatin tablet (cat. number H20051407, specification: 10 mg/tablet) was purchased from Pfizer Pharmaceutical Co., Ltd. (Dalian, China).

### 2.3. Experimental Design

ApoE^−/−^ mice are one of the most commonly used animal models of atherosclerosis in the world; the aortic atherosclerotic lesion may appear spontaneously or may be induced by a western-type diet in a short time [25]. It is generated on the gene background of C57BL/6J mice. So, we used ApoE^−/−^ mice as the model and C57BL/6J mice as the normal control. C57BL/6J mice were fed with a normal diet and designated as the control group (*n* = 10). ApoE^−/−^ mice were fed with a western-type diet (21% fat and 0.15% cholesterol, cat. number H10141, Beijing Huafukang Bioscience Co., Ltd.) for 6 weeks and then were divided into a model group (*n* = 10), BSKS group (*n* = 10), and atorvastatin group (*n* = 10). Mice in the model group were given 0.3 ml of isopycnic sterile distilled water by gavage, mice in the BSKS group were given 1365 mg/kg BSKS solution by gavage (human equivalent dose: 128 mg/kg·d), and mice in the atorvastatin group were given 3 mg/kg atorvastatin solution by gavage (human equivalent dose: 0.28 mg/kg·d). BSKS and atorvastatin solution preparation: BSKS tablets and atorvastatin tablets were crushed and ground by pestle and then were moved to a tube and dissolved in 0.3 ml isopycnic sterile distilled water for each mouse.

### 2.4. Tissue Collection and Weight Record

After 6 weeks of treatment, mice were anesthetized by injecting 10% chloral hydrate intraperitoneally, and body weights of all mice were recorded, and blood, liver, white adipose tissues, and aorta were collected for detection. Liver and white adipose tissues (epididymal fat pads) were dissected carefully from mice and placed in a physiological saline. They were dried with a filter paper and their weights were recorded and compared to the mice's own body weights. The ratio of the liver weight to the body weight and the ratio of the white adipose tissue weight to the body weight were used to evaluate body fat deposition after being fed with a western-type diet.

### 2.5. Hematoxylin and Eosin Staining

Fixed aortic roots (section from the aortic valve to the descending thoracic aorta) were dehydrated and embedded in paraffin. Five-micrometer cross sections were prepared and stained with Hematoxylin and Eosin. The areas of aorta and plaque were measured by two independent observers using the image analysis software Image-Pro Plus 6.0 (Media Cybernetics, Inc., Rockville, MD, USA), and then the atherosclerotic lesion size and relative lesion area of the aortic root were measured and calculated.

### 2.6. Blood Lipids Detection

Blood was collected from the mice's eyes (orbital canthus venous plexus) using a capillary glass tube. 0.1 ml of whole blood can be acquired each time. Blood was centrifuged for 15 min at a rate of 3000 rpm. Serum levels of total cholesterol (TC) and triglyceride (TG) (cats. numbers A111-1 and A110-1, Nanjing Jiancheng Bioengineering Institute, Nanjing, China) were detected by the single reagent COD-PAP method. Low-density lipoprotein cholesterol (LDL-C) and high-density lipoprotein cholesterol (HDL-C) (cats. numbers A113-1 and A112-1, Nanjing Jiancheng Bioengineering Institute, Nanjing, China) were detected by the double reagent direct method. A colorimetric analysis was executed using a microplate reader (type: Infinite M200 PRO, Tecan, Männedorf, Switzerland).

### 2.7. Enzyme-Linked Immunosorbent Assay (ELISA)

Levels of the following proteins in blood were measured according to the manufacturer's instructions: interleukin-1 beta (IL-1*β*), interleukin-10 (IL-10), monocyte chemotactic protein-1 (MCP-1), vascular cell adhesion molecule-1 (VCAM-1), and adiponectin (using mouse-specific ELISA kits (cats. numbers SEA563Mu, SEA056Mu, SEA087Mi, SEA547Mu, and SEA605Mu, Wuhan USCN Business Co., Ltd., Wuhan, China)). Briefly, samples were incubated for 30 min at 37°C and then were exposed to biotin-conjugated detection antibody and streptavidin-HRP, respectively, for 60 min at 37°C. A stabilized chromogen and stop solution were added to terminate the reaction, and then plates were read at 450 nm (OD values) within 2 hours using a spectrophotometer.

### 2.8. Western Blot Analysis

The aortas were dissected from mice and placed in Eppendorf tubes and immersed in liquid nitrogen to snap-freeze them. Five micrograms of the aorta was added with 300 *μ*l of ice-cold lysis buffer to homogenize, and then constant agitation was maintained for 2 h at 4°C. The tube was centrifuged for 20 min at 12,000 rpm at 4°C in a microcentrifuge, and then the supernatant was aspirated in a fresh tube and a protein quantification assay was performed. Equal amounts of protein (30 *μ*g protein/lane) were electrophoresed using 8–12% SDS-polyacrylamide gels in a Tris/HCl buffer system, followed by electrophoretic transfer to a PVDF microporous membrane, and incubated with the following primary antibodies: anti-adiponectin antibody (1 : 1000, cat. number 2789  s, Cell Signaling Technology), anti-adiponectin receptor 1 (AdipoR1) antibody (1 : 1000, cat. number ab126611, Abcam), anti-adiponectin receptor 2 (AdipoR2) antibody (1 : 1000, cat. number sc-514045, Santa Cruz), anti-Toll-like receptor 4 (TLR4) antibody (1 : 1000, cat. number 14358 s, Cell Signaling Technology), and anti-NF-*κ*B p65 antibody (1 : 1000, cat. number 8242 s, Cell Signaling Technology). After incubation overnight at 4°C, membranes were probed with secondary HRP-labeled IgG H&L antibodies and washed with phosphate-buffered saline three times. Specific bands of target proteins were visualized by chemiluminescence. All bands were analyzed semiquantitatively with ImageJ software (National Institutes of Health, Bethesda, Maryland, USA).

### 2.9. Statistical Analysis

Data were expressed as mean ± SD. A statistical analysis was performed using one-way analysis of variance (ANOVA) followed by Fisher's Least Significant Difference test for multiple comparisons. All analyses were performed using the Statistical Package for the Social Sciences (SPSS) version 11.5 (SPSS Inc., Chicago, IL, USA). *P* < 0.05 or 0.01 (two-sided) was regarded as statistically significant.

## 3. Results

### 3.1. BSKS Reduced Atherosclerotic Lesion in ApoE^−/−^ Mice

**Figure 1:**
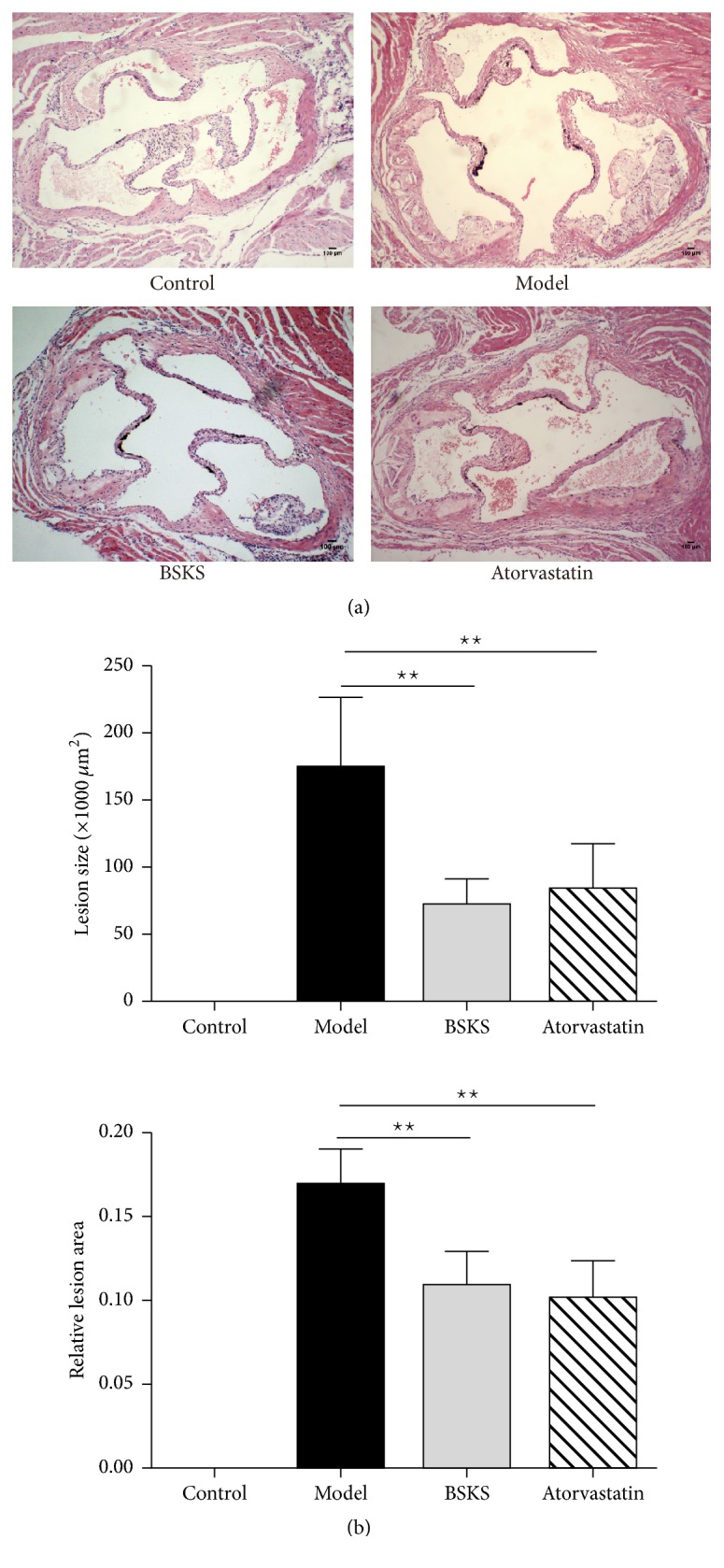
Effect of BSKS on the atherosclerotic lesion in ApoE−/− mice. (a) Representative photomicrographs of Hematoxylin and Eosin staining in the aortic root of male ApoE−/− mice treated with BSKS or atorvastatin. (b) The quantitative comparison of atherosclerotic lesion size and relative lesion area between drug treatment groups and model group (*n* = 10). Data are shown as mean ± standard deviation and compared by one-way analysis of variance followed by Fisher's Least Significant Difference test for individual comparisons. ^⋆⋆^*P* < 0.01.

To investigate the effect of BSKS on the development of atherosclerosis, ApoE^−/−^ mice fed with western-type diet were treated with BSKS for 6 weeks. The effect of BSKS on atherosclerosis was compared to positive drug atorvastatin. During the experiments, no adverse effect could be ever observed. The aortic root was used for the evaluation of atherosclerosis. Paraffin sections of the aortic root were stained with Hematoxylin and Eosin and analyzed quantitatively (Figure 1). The aortic root of ApoE^−/−^ mice showed thickened tunica intima compared to WT mice. BSKS and atorvastatin showed 58.62% and 51.72% reduction in the size of atherosclerotic lesion (72.02 × 10^3^ ± 19.10 × 10^3^ *μ*m^2^ in BSKS, 84.43 × 10^3^ ± 32.90 × 10^3^ *μ*m^2^ in atorvastatin versus 174.76 × 10^3^ ± 51.55 × 10^3^ *μ*m^2^ in model) and also showed significant reduction in the relative lesion area (*P* < 0.01). These results suggested that BSKS showed similar effect on atherosclerosis with atorvastatin.

### 3.2. BSKS Reduced Visceral Adipose Content and Decreased Blood Lipids Level

**Figure 2:**
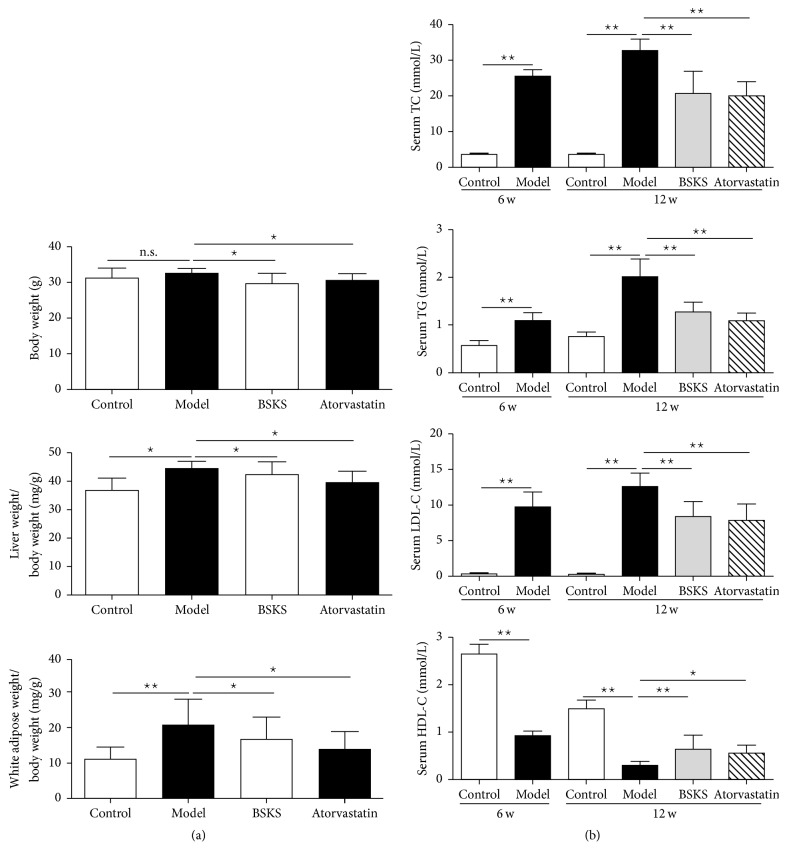
Effect of BSKS on visceral adipose weight and blood lipids in ApoE^−/−^ mice. (a) The body weight, liver weight, and white adipose weight of mice were recorded and compared between drug treatment groups and model group (*n* = 10). (b) The serum TC, TG, LDL-C, and HDL-C of ApoE^−/−^ mice were detected and compared between drug treatment groups and model group (*n* = 10). Data are shown as mean ± standard deviation and compared by one-way analysis of variance followed by Fisher's Least Significant Difference test for individual comparisons. _ _^n.s.^*P* > 0.05; _ _^⋆^*P* < 0.05; _ _^⋆⋆^*P* < 0.01.

To further prove the antiatherosclerotic effect of BSKS, we next evaluated visceral adipose accumulation and blood lipids level (Figure 2). We found that BSKS and atorvastatin significantly decreased the body weight of ApoE^−/−^ mice fed with western-type diet (*P* < 0.05). What is more, they also reduced the ratio of liver weight to body weight and the ratio of white adipose tissue to body weight (*P* < 0.05). ApoE^−/−^ mice fed with a western-type diet for 6 weeks had significantly increased serum TC, TG, and LDL-C and decreased HDL-C (*P* < 0.01, compared with WT mice). After drug treatment for 6 weeks, we found that BSKS showed a similar lipid-lowering effect compared to atorvastatin, which reduced serum TC, TG, and LDL-C and increased HDL-C significantly (*P* < 0.01). These results provided evidence that the antiatherosclerosis effect of BSKS might be due to improving blood lipids metabolism.

### 3.3. BSKS Promoted the Adiponectin-Dependent Anti-Inflammatory Pathway

**Figure 3:**
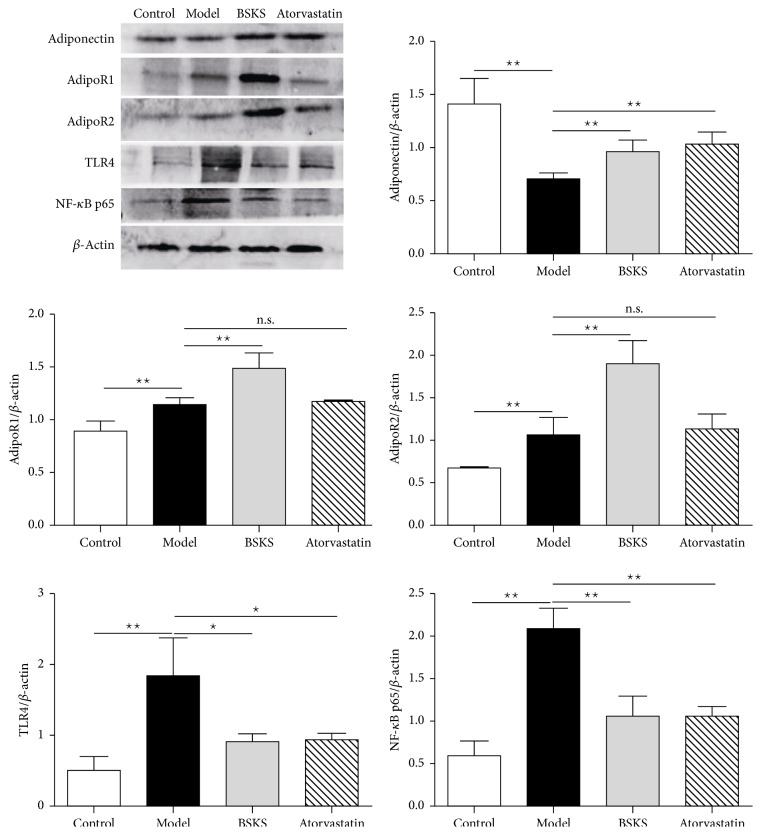
Effect of BSKS on the expression of adiponectin and its receptors as well as TLR4 and NF-*κ*B p65 in the atherosclerotic aorta of ApoE^−/−^ mice. The expressions of adiponectin, AdipoR1, AdipoR2, TLR4, and NF-*κ*B p65 in the atherosclerotic aorta of ApoE^−/−^ mice were detected by western blot. The expressions of adiponectin, AdipoR1, AdipoR2, TLR4, and NF-*κ*B p65 were quantitatively compared between drug treatment groups and model group (*n* = 3). Data are shown as mean ± standard deviation and compared by one-way analysis of variance followed by Fisher's Least Significant Difference test for individual comparisons. _ _^n.s.^*P* > 0.05; _ _^⋆^*P* < 0.05; _ _^⋆⋆^*P* < 0.01.

To explore the molecular mechanism of BSKS against atherosclerosis, we performed western blot analysis of atherosclerotic aorta (Figure 3). We found that BSKS and atorvastatin increased the expression of adiponectin in atherosclerotic aorta (*P* < 0.01). BSKS but not atorvastatin promoted the expression of AdipoR1 and AdipoR2 (*P* < 0.01). Atherosclerosis-mediated inflammatory response promoted the expression of inflammatory receptor TLR4 and inflammatory signaling NF-*κ*B significantly (*P* < 0.01). BSKS and atorvastatin exerted significant inhibition of TLR4 and NF-*κ*B (*P* < 0.05  or  0.01). The inflammatory inhibition effect of BSKS might be due to the promotion of adiponectin and its receptors.

### 3.4. BSKS Decreased Proinflammatory Mediators and Increased Anti-Inflammatory Mediators

**Figure 4:**
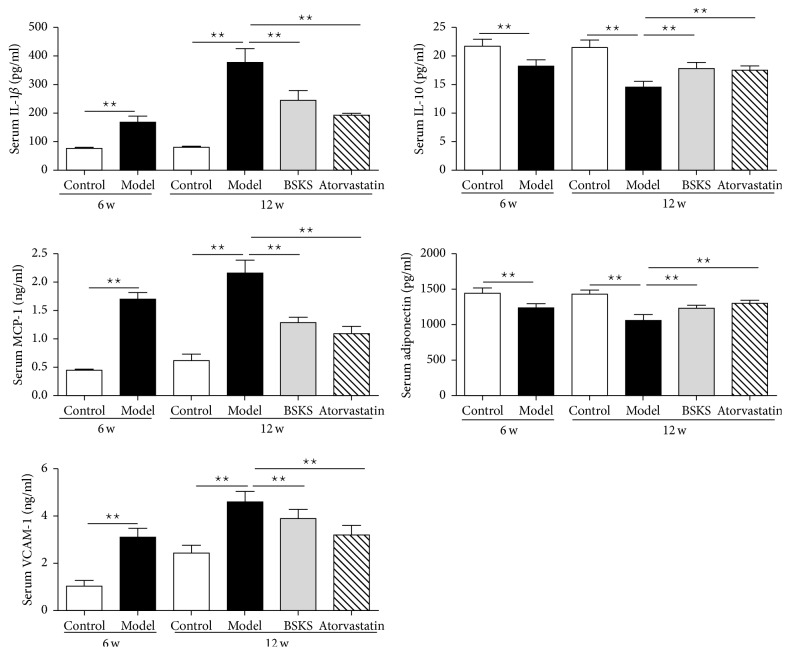
Effect of BSKS on the inflammatory mediators in blood of ApoE^−/−^ mice. The serum levels of proinflammatory mediators, including IL-1*β*, MCP-1, and VCAM-1, and anti-inflammatory mediators, including IL-10 and adiponectin, were detected by enzyme-linked immunosorbent assay and quantitatively compared between drug treatment groups and model group (*n* = 10) at 6 weeks or 12 weeks. Data are shown as mean ± standard deviation and compared by one-way analysis of variance followed by Fisher's Least Significant Difference test for individual comparisons. _ _^⋆⋆^*P* < 0.01.

As the inflammatory signaling pathway was inhibited by BSKS, we wanted to find out whether BSKS could affect the downstream inflammatory mediators. We performed ELISA to detect their levels in serum (Figure 4). The results showed that ApoE^−/−^ mice fed with a western-type diet for 6 weeks had increased levels of serum proinflammatory mediators (*P* < 0.01), including IL-1*β*, MCP-1, and VCAM-1, and decreased levels of serum anti-inflammatory mediators (*P* < 0.01), such as IL-10 and adiponectin. After drug treatment for 6 weeks, we found that both BSKS and atorvastatin decreased the levels of proinflammatory mediators and increased the levels of anti-inflammatory mediators significantly (*P* < 0.01). The above results indicated that BSKS not only inhibited inflammatory signaling protein but also regulated downstream inflammatory mediators.

## 4. Discussion

This study showed that BSKS reduced atherosclerotic lesions in the ApoE^−/−^ mice model. The mechanism of BSKS was involved in reducing visceral adipose content, declining blood lipids, increasing the expression of adiponectin and its receptor, and reducing the expression of TLR4 and NF-*κ*B in atherosclerotic aorta, as well as promoting anti-inflammatory mediators release and inhibiting proinflammatory mediators production in the blood. This study provides potential molecular mechanisms for the clinical application of BSKS in cardiovascular diseases.

Ischemic vascular diseases are mostly correlated with atherosclerotic lesions [23], while obesity shares a great many risk factors with atherosclerosis, suggesting some common mechanisms in these diseases, such as a proatherogenic lipid profile [26], increased production of proinflammatory cytokines [27], and prevalence of endothelial dysfunction [28]. Visceral adipose tissue, but not subcutaneous adipose tissue, is especially associated with increased cardiovascular risk [29]. Evidence has shown that visceral adipose-related inflammation promotes the development of atherosclerosis in ApoE^−/−^ mice [30]. In our study, we found that western-type diet increased the weight of the liver and white adipose tissues, and BSKS treatment decreased the ratio of visceral adipose weight to body weight. What is more, BSKS administered for 6 weeks significantly reduced the levels of TC, TG, and LDL-C in the blood and increased the level of HDL-C, and these effects were similar to those of atorvastatin. These results suggested that BSKS reduced atherosclerotic lesions by decreasing visceral adipose-related inflammation and decreasing blood lipids.

Adiponectin, a hormone secreted by adipocytes, plays an important role in protecting against the development of atherosclerosis [31, 32]. The underlying mechanism of adiponectin against atherosclerosis is due to inhibition of NF-*κ*B signaling in monocytes/macrophages and in endothelial cells [33]. In addition, adiponectin could reduce endothelial cell apoptosis and promote nitric oxide production through adenosine 5′-monophosphate- (AMP-) activated protein kinase (AMPK) signaling pathway [34]. Finally, adiponectin inhibits smooth muscle cells proliferation and migration to reduce foam cells formation by activating the AMPK signaling pathway [35]. Here, in our study, adiponectin and its receptors in the atherosclerotic aorta and adiponectin in the blood were upregulated by BSKS treatment, while the expression of TLR4 and NF-*κ*B p65 in the aorta was inhibited by BSKS, followed by the suppression of proinflammatory mediators, including IL-1*β*, MCP-1, and VCAM-1. These results suggested that the antiatherosclerotic effect of BSKS might be involved in promoting anti-inflammatory mediators and inhibiting the inflammatory response via TLR4 and NF-*κ*B p65 signaling pathway. On the other hand, cholesterol accumulation in macrophages promotes inflammatory responses, and activated inflammatory signaling results in cholesterol accumulation in foam cells and further amplifies inflammatory responses [36], so there is a cross talk between cholesterol accumulation and inflammatory responses. The reduction of atherosclerotic lesion and improvement of blood lipids metabolism by BSKS might be the reason for the inhibition of inflammatory signaling pathway and proinflammatory mediators.

In conclusion, this study indicated that the development of atherosclerosis was able to be alleviated by BSKS in vivo. The underlying mechanism of BSKS was associated with improving blood lipids metabolism, promoting adiponectin and its receptors in atherosclerotic lesions, and inhibiting the inflammatory signaling pathway. However, further studies are required to elucidate which active ingredient of BSKS plays the major role in suppressing atherosclerosis.
